# Tracking Antimicrobial Resistance in *Salmonella* via Poultry Supply Chains, Human Clinical Samples, and Environmental Reservoirs

**DOI:** 10.3390/foods15030410

**Published:** 2026-01-23

**Authors:** Diana M. Álvarez-Espejo, Diego Fredes-García, Constanza Díaz-Gavidia, Sebastián Gutiérrez, Rocio Barron-Montenegro, Francisca P. Álvarez, Rodrigo Constenla-Albornoz, Vivien Cadet-Arenas, Angélica Reyes-Jara, Jorge Olivares-Pacheco, Elton Burnett, Rebecca L. Bell, Magaly Toro, Jianghong Meng, Patricia García, Andrea I. Moreno-Switt

**Affiliations:** 1 Escuela de Medicina Veterinaria, Pontificia Universidad Católica de Chile, Santiago 7820436, Chile; dlalvarez@uc.cl (D.M.Á.-E.); diegfredes@gmail.com (D.F.-G.); constanza.diaz@uc.cl (C.D.-G.); rnbarron@uc.cl (R.B.-M.); fpia.alvarez@gmail.com (F.P.Á.); vpcadet@uc.cl (V.C.-A.); 2 Escuela de Enfermería, Facultad de Medicina, Pontificia Universidad Católica de Chile, Santiago 7820436, Chile; 3 Laboratorio de Microbiología y Probióticos, Instituto de Nutrición y Tecnología de Los Alimentos (INTA), Universidad de Chile, Santiago 7830490, Chile; sebastiangutierrez@ug.uchile.cl (S.G.); areyes@inta.uchile.cl (A.R.-J.); 4 Laboratorio de Salud Pública, Ambiental y Laboral, SEREMI Salud, Región de Valparaíso, Valparaíso 2340000, Chile; rodrigo.constenla@gmail.com; 5 Grupo de Resistencia Antibacteriana en Bacterias Patógenas y Ambientales (GRABPA), Instituto de Biología, Pontificia Universidad Católica de Valparaíso, Valparaíso 2340000, Chile; jorge.olivares@pucv.cl; 6 Institute of Parasitology, McGill University, Montreal, QC H9X 3V9, Canada; elton.burnett@mail.mcgill.ca; 7 Center for Food Safety and Applied Nutrition, Food and Drug Administration, College Park, MD 20740, USA; rebecca.bell@fda.hhs.gov; 8 The Joint Institute for Food Safety and Applied Nutrition (JIFSAN), The University of Maryland, College Park, MD 20740, USA; mtoroiba@umd.edu (M.T.); jmeng@umd.edu (J.M.); 9 Departamento de Laboratorios Clínicos, Facultad de Medicina, Pontificia Universidad Católica de Chile, Santiago 7820436, Chile; pgarciacan@uc.cl

**Keywords:** *S.* Infantis, *S.* Heidelberg, poultry, chicken meat, pESI-like, One Health

## Abstract

The global dissemination of multidrug-resistant (MDR) *Salmonella* through the international food trade poses a major One Health concern. We used whole-genome sequencing to characterize *Salmonella* isolates from poultry meat sold in Chile, including domestic and imported products from Brazil and Argentina. Sixty-one *Salmonella* isolates were recovered from poultry meat; *S.* Infantis predominated (59%), followed by *S.* Heidelberg. Among *S.* Heidelberg from imported-meat poultry, 92% carried the *bla*_CMY-2_ gene, conferring resistance to β-lactams. Given the predominance of *S.* Infantis in poultry meat, we performed an additional in-depth genomic analysis of 73 *S.* Infantis isolates obtained from poultry meat (*n* = 32), surface water (*n* = 30), and human clinical cases (*n* = 11). Across sources, phenotypic resistance to ciprofloxacin and third-generation cephalosporins reached 93% and 70%, respectively, and MDR (≥3 antimicrobial classes) occurred in 71% of isolates, largely associated with *bla*_CTX-M-65_ and *gyrA* mutations. The pESI (plasmid of emerging *S.* Infantis)-like plasmid, harboring antimicrobial resistance and virulence genes, appeared in 94% of isolates. Phylogenetic analyses showed close genetic relationships among food, environmental, and clinical isolates, suggesting potential transmission through contaminated poultry meat or water. These findings emphasize the emergence of MDR *S.* Infantis in Chile and underscore the need for integrated One Health surveillance and prudent antimicrobial use to mitigate foodborne AMR risks.

## 1. Introduction

*Salmonella* is a prevalent foodborne pathogen primarily transmitted through contaminated food or contact with infected animals. In Latin America, non-typhoidal *Salmonella* is frequently reported in foods, including poultry products [1]. Chile represents a relevant setting because poultry meat is supplied by both domestic production and imports primarily from Brazil and Argentina [2], creating opportunities for cross-border dissemination of circulating serovars and antimicrobial resistance determinants [3,4]. The global burden of salmonellosis is estimated at 94 million cases of non-typhoid *Salmonella* and 155,000 associated deaths each year [5,6,7]. Therefore, *Salmonella* remains a major cause of foodborne illness and outbreaks worldwide [7]. Approximately 2600 *Salmonella* serovars have been identified in humans [8], animals [9], food [10], and environmental sources [11]. However, serovar diversity is a dynamic characteristic, with the prevalence of specific serovars varying regionally and over time [12,13].

Notably, international trade of poultry meats is one of the ways that predominant serovars are disseminated across different countries or regions [4]. Several *Salmonella* serovars, such as *S.* Enteritidis, *S.* Typhimurium, *S.* Kentucky, *S.* Heidelberg*,* and *S.* Infantis, are frequently associated with poultry products and pose a risk for human transmission [3]. In parallel, antimicrobial resistance (AMR) is an increasing global threat, including in foodborne pathogens disseminated through trade networks [4]. AMR is an escalating global health threat, and foodborne pathogens such as *Salmonella* increasingly carry determinants of resistance to clinically important antimicrobials [3].

Reports of salmonellosis due to multidrug-resistant (MDR) *S*. Infantis strains have increased over the past decade. This serovar has been reported in MDR form across multiple regions worldwide, including Europe, the Americas, and Asia [14,15,16]. Resistant isolates were identified in humans, food samples, and animal samples, highlighting poultry as the primary reservoir [17]. Studies indicate that *S.* Infantis isolates have shown resistance to several antimicrobial classes such as ciprofloxacin, cephalosporins, aminoglycosides, and tetracyclines [17]. Emergent MDR *S.* Infantis strains are typically associated with the pESI (plasmid of emerging *S.* Infantis), a ~300 kb megaplasmid [14,18]. The pESI megaplasmid is characteristic of certain lineages of *Salmonella* Infantis and harbors a wide range of genes associated with bacterial pathogenicity and resistance to multiple antibiotics [19,20].

Our research questions were: What are the serovar distribution and antimicrobial resistance profiles of *Salmonella* isolated from poultry meat sold in Chile (domestic and imported)? How do these relate to the phenotypic/genotypic AMR patterns? What is the genomic relatedness among *S.* Infantis isolates from poultry meat, surface water, and human clinical cases? Then, the present study aimed to investigate the genomic and antimicrobial resistance profiles of *Salmonella* isolates from poultry meat sold in Chile, including both domestic products and imports from Brazil and Argentina, using whole-genome sequencing (WGS) and antimicrobial susceptibility testing. After identifying *Salmonella* Infantis as the predominant serovar in poultry meat, we expanded our analysis to include isolates from human clinical cases and surface waters to assess genomic relatedness and AMR determinants across sources. By integrating genomic and phenotypic data, we highlight the value of integrated surveillance to understand the emergence and dissemination of high-risk MDR *Salmonella*.

## 2. Materials and Methods

### 2.1. Sample Collection and Salmonella Isolation

Poultry sampling was carried out by the Secretariat of Public Health and the Environmental Laboratory and Occupational Health of SEREMI Valparaíso in 110 locations across the Valparaíso region in 2018. Sampling sites included food retail outlets, supermarkets, and containers or storage facilities used for imported products. Multiple samples were collected at each sampling site. These sites were selected based on risk assessments, import records, and reports on foodborne illnesses [21,22]. Considering foodborne disease reports [22], the Regional Health Authority (SEREMI) of Valparaíso prioritized sampling of poultry meat. Sampling sites were selected based on SEREMI risk assessment studies (not publicly available), imports entering through the region, and records of food-related complaints and/or foodborne intoxications, encompassing food retail outlets, supermarkets, and containers or storage facilities used for imported products. A total of 61 isolates were obtained from meat samples, comprising chicken (*n* = 52) and turkey (*n* = 9) (Supplemental Table S1A). Isolates from meat originated from international trucks, company branches (specifically those involved in chicken and turkey meat sales), factories, and retail outlets.

Poultry samples were analyzed with the validated Association Française de Normalisation (AFNOR) BIO-12/16-09/05 method, using Vitek Immuno Diagnostic Assay System–Positive Control (VIDAS PC) Easy for the detection of *Salmonella* spp. (BioMérieux, Lombard, IL, USA). Positive samples were confirmed in accordance with the International Organization for Standardization (ISO 66579-1:2017) [23] standard guidelines, utilizing selective agars XLD (Xylose-Lysine_Deoxycholate) (Becton, Dickinson Company, Sparks, MD, USA, CHROMIDÒ *Salmonella* Elite (BioMériux, Lombard, IL, USA), and Brilliant Green Sulfa agar Becton, Dickinson Company, Sparks, MD, USA. Five colonies were selected for confirmation using the Analytical Profile Index (API) 20E microbial identification kit (BioMériux, Lombard, IL, USA), according to the manufacturer’s instructions. One of the five colonies identified as belonging to the *Salmonella* genus was selected for serological identification at the Public Health Institute of Chile, which serves as the national reference laboratory. Only one colony per positive sample was selected for serological identification; therefore, within-sample heterogeneity may not have been captured.

Clinical isolates (*n* = 11) were obtained by the UC-CHRISTUS Health Network laboratory. These strains were isolated from urine, stool samples, peritoneal fluid, and abdominal wounds between 2016 and 2021 in the Santiago Metropolitan Region (Supplemental Table S1B). The samples were processed following protocols established by the UC-CHRISTUS Health Network Laboratory [24]. Briefly, samples were transported in Cary Blair medium (Eurotubo^®^, I.A.S.A, Parets del Vallès, Spain) and plated on Hektoen agar (BioMérieux, Ha, MO, USA), MacConkey (BioMérieux, Ha, MO, USA), and selenite broth (BioMérieux, Ha, MO, USA, with reseeding on Hektoen agar after incubation for 16 h at 35° C. *S.* Infantis isolates were searched and identified by conventional methods, according to the procedures outlined in the Manual of Clinical Microbiology [25]. Clinical isolates were obtained from a single clinical network (UC-CHRISTUS) in the Santiago Metropolitan Region and therefore represent a convenience sample, which may introduce sampling bias and limit the generalizability of clinical findings to the national level.

Surface water isolates from the Maipo and Mapocho rivers, located in Chile’s central region, were analyzed from April 2019 to February 2020. A total of 300 samples were collected from the Mapocho River and 240 samples from the Maipo River during the sampling period from April 2019 to February 2020 [26]. The isolation process was carried out using a modified version of the Food and Drug Administration-Bacteriological Analytical Manual (FDA-BAM) protocol, as described in Toro et al., 2022 [26]. Positive samples and more details available in Huang et al., 2024 [11]. From these samples, 41 isolates identified as *S.* Infantis (using whole-genome sequencing) were selected for this study (Supplemental Table S1C).

### 2.2. Whole-Genome Sequencing

A total of 113 isolates were sequenced, comprising 61 isolates from poultry meat, 41 from water sources, and 11 from clinical cases (Supplemental Table S1A–C).

Genomic DNA from poultry meat isolates was extracted using the Cultured Cells DNA Kit on a Maxwell RSC-48 Instrument (Promega, Madison, WI, USA). Libraries were constructed with the Illumina DNA Prep kit (Illumina, San Diego, CA, USA) on the Sciclone G3 NGSx iQ Workstation (PerkinElmer, MA, USA), and sequencing was conducted by the Florida Department of Health using the Illumina NextSeq 2000 platform with 300-cycle paired-end chemistry (Illumina, San Diego, CA, USA) and the NextSeq 1000/2000 P2 reagents (Illumina Inc., San Diego, CA, USA). All reads were submitted in FASTQ format to the NCBI Sequence Read Archive (SRA). Genome assemblies were generated using SPAdes v3.14.0.

For clinical isolates, DNA extraction was performed using the DNeasy Blood & Tissue kit (Qiagen, Redwood City, CA, USA) following the manufacturer’s instructions. Sequencing was carried out at the Microbial Genome Sequencing Center (MiGS-SeqCenter, Pittsburgh, PA, USA), and raw reads were assembled through the EnteroBase platform [27]. Genome annotation was conducted with Prokka v1.13, and core genome analyses were performed using Roary v3.12.0 [28,29]. Surface water isolates were sequenced and processed following the same protocol used for poultry meat isolates, at the Human Foods Program of the US Food and Drug Administration (FDA), as previously described in Chen et al., 2024 [30]. All genome assemblies are publicly available through the NCBI Pathogen Detection database (Supplemental Table S1).

### 2.3. Sequence Analysis

Genome sequencing was employed to predict *Salmonella* serovars for each meat isolate, stratified by country of origin and source, to determine the serovar distribution. Serotype prediction was performed on assembled genomes using the SeqSero2 v1.2.1. Among identified serovars, Heidelberg and Infantis were the most prevalent and were therefore selected for downstream analysis. Antibiotic resistance genes and point mutations in these isolates were identified using AMRFinderPlus v3.11.4, ABRicate v.0.8.10, and ResFinder.

Roary v3.12.0 was used for reference-free generation of the core genome alignment. Core genome SNPs (cgSNPs) were identified using snp-sites v2.5.1. Pairwise SNP distances were computed as the number of differing nucleotide positions across the cgSNP alignment (excluding sites with gaps or ambiguous bases). Isolates differing by 0–5 SNPs were considered clonal; one representative isolate per clonal group was retained for downstream analyses. Phylogeny reconstruction was performed using IQ-TREE v1.6.11 with the maximum likelihood option, and phylogenies were midpoint-rooted and annotated using the Interactive Tree of Life (iTOL) v5 [31]. Cytoscape 3.8.2 was used for the visualization of networks of Antimicrobial Resistance Genes (ARGs).

To investigate the emergence and dissemination of MDR *Salmonella* Infantis in Chile, we compared *S.* Infantis isolates collected between 2018 and 2021 from food (poultry meat from this study), human clinical cases, and environmental (surface water) sources (Supplemental Table S1).

We screened whole-genome sequencing of the poultry meat, and surface water isolates to identify and exclude clonal strains as described above. Based on this SNP distance analysis, a non-redundant set of 73 *S.* Infantis isolates were selected for the analysis, including isolates from poultry meats (*n* = 32), surface water (*n* = 30), and human clinical (*n* = 11). These isolates were subjected to in-depth genotypic and phenotypic characterization to assess the potential public health threat posed by emerging MDR *Salmonella* Infantis strains in Chile (see Supplemental Table S2 for details).

### 2.4. Plasmid Analysis

Plasmid replicons were identified using ABRicate v.0.8.10 along with the PlasmidFinder database. A representative pESI-like plasmid was obtained from surface water isolate FA0496 using the PlasmidSPAdes tool (Galaxy Version 4.2.0) [32]. Because a single representative pESI-like plasmid (FA0496) was reconstructed from short-read data, this sequence may not capture the full diversity of pESI-like plasmids across isolates (e.g., structural variation or gene content differences), and plasmid features should be interpreted in this context. To complement and generate a consensus sequence, the plasmid sequence was aligned against the complete genomic sequence of the same isolate using Mauve Contig Mover in Geneiuos Prime v2023.1. Subsequently, plasmid sequences were annotated with prokka v1.14.6 [28] on the GalaxyTrakr platform. Geneious Prime v2023.1 was utilized for the generation, editing, and visualization of the plasmid sequence.

Using the genomic data, we calculated the proportion of *S.* Infantis isolates carrying horizontally acquired resistance genes, chromosomal mutations associated with resistance to each antibiotic class, and the presence of the pESI-like megaplasmids. A diagram representing the detected AMR genes and chromosomal mutations was constructed using R software (version 4.0.4, The R Foundation, Vienna, Austria) to identify genes found in a single source (e.g., only human isolates) or shared across multiple sources.

### 2.5. Antimicrobial Susceptibility Testing in S. Infantis

Antimicrobial susceptibility testing was performed on *S.* Infantis isolates from poultry meat (*n* = 32), surface water (*n* = 30), and human clinical (*n* = 11) isolates (see Supplemental Table S2 for details on this selection) using the minimum inhibitory concentration (MIC) method following Clinical Laboratory Standards Institute (CLSI) guidelines [33]. Categorical interpretations were confirmed using CLSI M100 and were unchanged [33]. Isolates were analyzed on Mueller Hinton II agar (MH, Difco, Detroit, MI, USA), and nineteen antibiotics were tested: ampicillin, ampicillin-sulbactam, cefazolin, cefuroxime, cefotaxime, ceftazidime, cefixime, cefepime, cefotaxime/clavulanate, ceftazidime/clavulanate, piperacillin/tazobactam, pefloxacin, ciprofloxacin, ertapenem, meropenem, imipenem, gentamicin, amikacin, trimethoprim-sulfamethoxazole; all antibiotics were obtained from Oxoid (Basingstoke, UK) [34] (Supplemental Table S3). Isolates classified as “intermediate” in the antimicrobial susceptibility test were treated as resistant for the purposes of this study [35]. This approach was used to report conservative estimates of non-susceptibility for epidemiological comparisons across sources and to avoid underestimating resistance/MDR in the absence of isolate-level clinical exposure or dosing information. The phenotypic test for Extended Spectrum Beta-lactamase (ESBL) production was performed according to CLSI 2025. We specifically examined susceptibility to cefotaxime and cefotaxime/clavulanate and ceftazidime and ceftazidime/clavulanate [33] (Supplemental Table S3).

The overall proportion of *S.* Infantis isolates exhibiting phenotypic resistance to each antibiotic tested was calculated as a percentage for isolates from poultry meat, human samples, and surface waters. Similarly, the proportion of *S.* Infantis isolates producing extended-spectrum beta-lactamases (ESBL) from these three sources was also expressed as percentages. Finally, the percentage of isolates classified as multidrug-resistant (MDR), defined as acquired resistance to at least one antibiotic in three or more antimicrobial categories, was calculated.

## 3. Results

### 3.1. Salmonella Isolates from Poultry Meat

**Figure 1:**
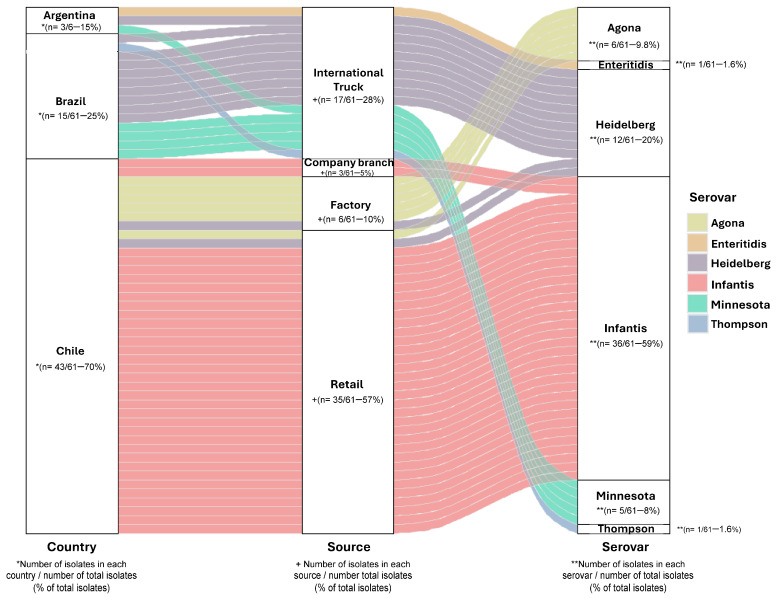
Origin and serovar of *Salmonella* isolates from poultry meat. Diagram of 61 chicken meat isolates by country of origin, source, and serovar predicted by the SeqSero2 program v1.2.1. * Number of isolates in each country/number of total isolates (% of total isolates); + Number of isolates in each source/number of total isolates (% of total isolates); ** Number of isolates in each serovar/number of total isolates (% of total isolates) (Information detailed in Supplemental Table S1).

A total of 61 *Salmonella* isolates were collected and analyzed in 2018 from poultry meat sampled in Chile, including domestically produced meats (70%; 43/61) and imported meats from Brazil (25%; 15/61) and Argentina (5%; 3/61) (Figure 1). Most of the isolates (57%; 35/61) were obtained from retail outlet samples, and *S.* Infantis was the most common serovar found (59%; 36/61) (Figure 1 and Table 1).

*S.* Heidelberg isolates from Brazil, Argentina, and Chile shared a conserved genotypic resistance profile, including *aac(6′)-IV*, *bla*_CMY-2_, *bla*_CMY-9_, *fos*A7, *sul*2, and *tet*(A) and chromosomal *gyrA* mutations S83F (Supplemental Figure S1A). All *S.* Infantis isolates shared a consistent resistance gene profile, including *aac-(3)-IV*, *acc-(6′)-IV*, *ant(3″)-IIa*, *aph(3′)-Ia*, *aph(4)-Ia*, *sul*1, *tet*(A), *floR*, *bla*_CTX-M-65_, *fos*A, *dfrA14*, and a g*yrA* mutation D87Y (Supplemental Figure S1B). Our data showed that in this 2018 poultry-meat isolate collection, *S.* Infantis was the most frequently detected serovar, particularly among isolates from retail outlets and company branches. We also detected *S.* Agona in turkey meat processed at the abattoir. *S.* Minnesota was detected only in imported products (Brazil and Argentina), and *S.* Heidelberg was recovered from both domestic and imported chicken, with the highest proportion observed among imports from Brazil.

### 3.2. Antimicrobial Susceptibility in Salmonella Infantis

**Figure 2:**
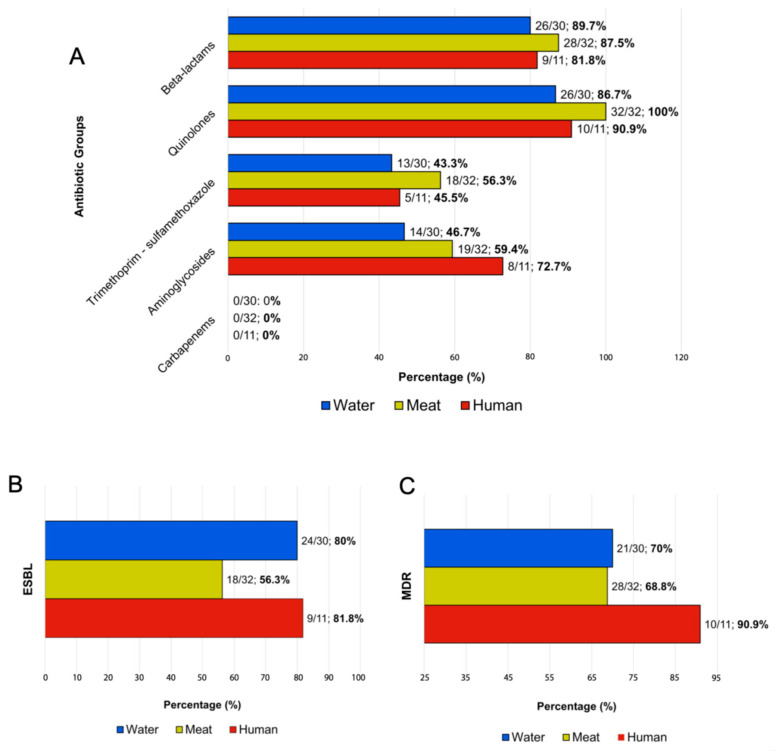
Frequency of antimicrobial-resistant phenotypic profiles of *Salmonella* Infantis isolate from humans, surface water, and poultry meat. Seventy-three isolates were analyzed for antimicrobial susceptibility. (**A**) Percentage of resistance to antibiotic classes tested. (**B**) Percentage of detection of Extended Spectrum Beta-lactamase (ESBL). (**C**) Percentage of multidrug resistance (MDR) isolates according to the source. Bars representing human isolates are in red (*n* = 11), surface water in blue (*n* = 30), and chicken meat in yellow (*n* = 32). Percentages are shown in bold.

The antimicrobial resistance of 73 *S.* Infantis isolates was evaluated against 19 antimicrobial agents. Isolates from human samples (*n* = 11) showed resistance to quinolones (91%; 10/11), beta-lactams (82%; 9/11), gentamicin (73%; 8/11), and trimethoprim/sulfamethoxazole (45%; 5/11) (Figure 2A and Table 2). Among poultry meat isolates (n = 32), resistance was observed against quinolones (100%; 32/32), beta-lactams (88%; 28/32), gentamicin (59%; 19/32), and trimethoprim/Sulfamethoxazole (56%; 18/32) (Figure 2A and Table 2). In surface water isolates, we identified resistance to quinolones (87%; 26/30), beta-lactams (80%; 24/30), gentamicin (50%; 15/30), and trimethoprim/sulfamethoxazole (40%; 12/30) (Figure 2A and Table 2). None of the tested isolates (*n* = 73) showed carbapenem resistance (Figure 2A).

Five isolates (7%, 5/73) (human = 1, surface water = 4) showed susceptibility to all the antibiotics examined (Supplemental Table S4). ESBL production was detected in 81% (9/11) of human isolates, 56% (18/32) of poultry meat, and 80% (24/30) of water isolates (Figure 2B). Furthermore, we observed multidrug-resistant isolates in all categories evaluated: human (90%; 10/11), poultry meat (68%; 28/32), and surface water (70%; 21/30) (Figure 2C).

### 3.3. Antibiotic Resistance Genes and Determinants in Salmonella Infantis

**Figure 3:**
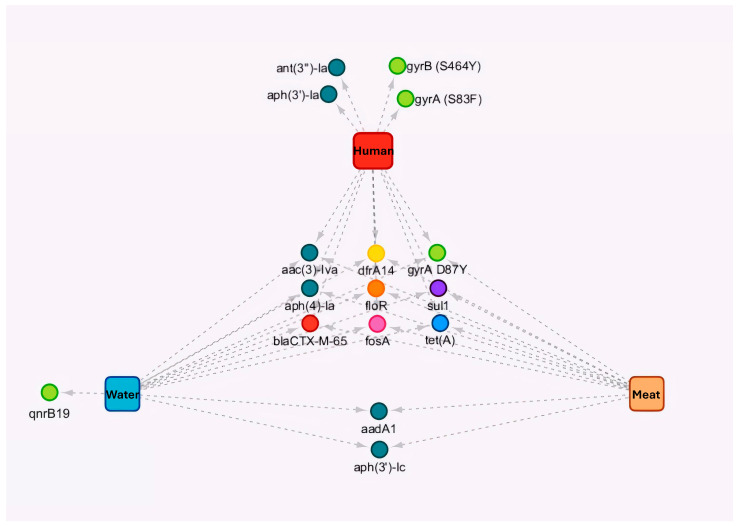
Diagram of the genes and chromosomal mutations detected in *Salmonella* Infantis isolates from humans, surface water, and poultry meat. Resistant determinants were detected in 73 *S.* Infantis isolates from humans (red, *n* = 11), surface water (blue, *n* = 30), and poultry meat (orange, *n* = 32), indicating the common genes and chromosomal mutations between sources and the resistance genes detected in each source. The circle color indicates the class of antibiotics, the arrows indicate the resistance genes present in each of the sources.

The most prevalent resistance genes in human, poultry meat, and surface water isolates were described in Table 3 and Supplementary Table S4. The analysis of *S.* Infantis genomes (*n* = 73) revealed the presence of eight resistance genes across isolates from all sources: *acc(3)-IV*, *dfrA14*, *aph(4)-la*, *floR*, *sul1*, *bla*_CTX-M-65_, *fosA*, *and tet(A)*. In addition, the *gyrA* D87Y mutation was identified across isolates from different sources (Figure 3 and Table 4). A single human clinical isolate did not carry resistance genes or plasmids (Supplementary Table S4) and corresponded to the only pan-susceptible clinical isolate in this dataset. Overall, MIC-based resistance patterns were consistent with the detected resistance determinants. In particular, quinolone non-susceptibility aligned with QRDR mutations (predominantly *gyrA* D87Y), while resistance to extended-spectrum cephalosporins was consistent with ESBL-associated genes (e.g., *bla*_CTX-M-65)_ (Table 2, Table 3 and Table 4). No major genotype–phenotype discrepancies were observed; notably, the only pan-susceptible clinical isolate lacked acquired resistance genes and plasmid replicons (Supplementary Table S4).

### 3.4. Salmonella Infantis Phylogeny

**Figure 4:**
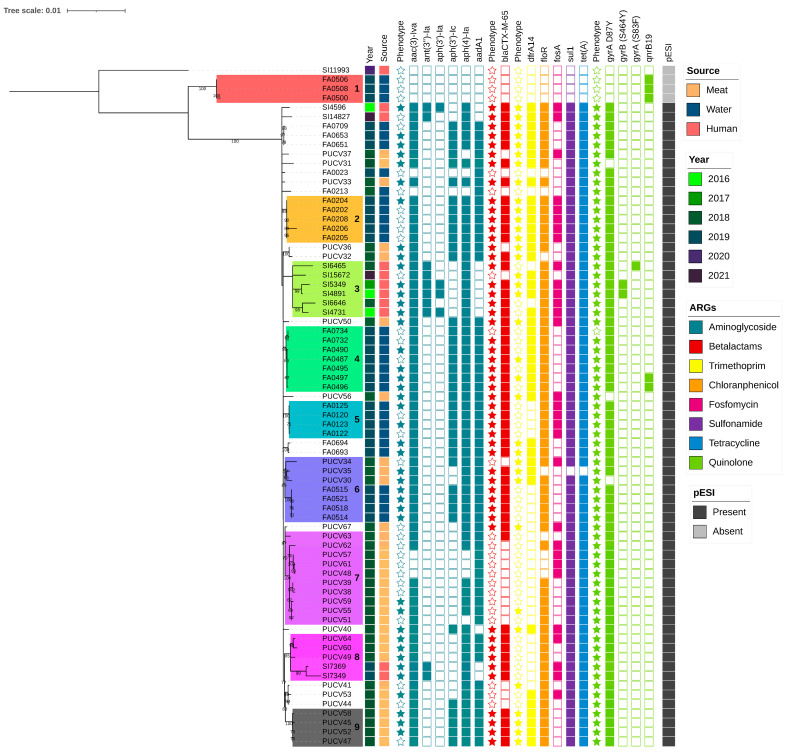
Phylogenetic tree based on core-genome Single Nucleotide Polymorphisms (cgSNPs) of 73 *Salmonella* Infantis strains from human, surface water, and poultry meat isolates. The legend indicates the source of origin, year of isolation, and presence of pESI plasmid. Additionally, antimicrobial resistance genes (ARGs) are indicated for each isolate. Stars indicate that the isolates exhibit phenotypic resistance to the antibiotics corresponding to the resistant genes grouped by antibiotic class (blue ocean color: aminoglycosides; red: beta-lactam; yellow: trimethoprim; orange: florfenicol; fuchsia: fosfomycin; violet: sulphonamides; blue, cerulean: tetracyclines; green lime: quinolones). The clusters described in the text are color coded and numbers were serially added from 1–9.

We analyzed the SNP-based phylogeny of 73 isolates of *S.* Infantis from humans, poultry meat, and surface water, identifying nine clusters with potential epidemiological importance (Figure 4). Clusters 1, 2, 4, and 5 consisted exclusively of surface water isolates; clusters 7 and 9 included only poultry meat isolates, while cluster 3 comprised only human clinical isolates (Figure 4).

Clusters from surface waters showed high intra-cluster genetic similarity; Cluster 1 (4–8 SNPs difference among isolates) was comprised of surface water isolates that lacked the pESI plasmid and only carried the AMR gene qnrB19. Clusters 2 (5–13 SNPs), and 5 (1–9 SNPs) were formed by Mapocho river isolates, while cluster 4 (2–8 SNPs) included isolates from both the Mapocho and Maipo rivers. Cluster 3 (23–68 SNPs) was composed of isolates from humans collected between 2016 and 2020. These isolates shared a consistent genotypic resistance profile and carried the pESI plasmid. Cluster 7 (0–17 SNPs) and Cluster 9 (0–4 SNPs) were composed of poultry meat isolates collected in 2018 and exhibited similar resistance to quinolones, beta-lactams, gentamicin, and trimethoprim/sulfamethoxazole. These isolates shared resistance genes including *aac(3)-IVa*, *aadA1*, *aph(4)-Ia*, *bla*_CTX-M-65_, *dfrA14*, *floR*, *sul1*, *tet(A)*, *gyrA*(D87Y), and harbored pESI.

Notably, clusters 6 and 8 included isolates from two sources (Figure 4). Cluster 6 (1–33 SNPs) revealed a distinct clade linking isolates from chicken meat and surface water isolates collected between 2018 and 2019. All these isolates carried the pESI plasmid, though resistance gene profiles differed slightly between sources, particularly for aminoglycosides and trimethoprim. Cluster 8 (6–70 SNPs) included five isolates from both poultry meat and human isolates collected in 2018 and 2019. These isolates shared resistance to quinolones, beta-lactams, gentamycin, and trimethoprim/sulfamethoxazole and carried *aac(3)-IVa*, *aadA1*, *aph(3′)-Ic*, *aph(4)-Ia*, *bla*_CTX-M-65_, *dfrA14*, *floR*, *sul1*, *tet(A)*, *gyrA*(D87Y), along with the pESI plasmid. Notably, within cluster 8, we identified two genetically identical human clinical isolates obtained from different areas of Santiago and at different time points in 2019.

### 3.5. Plasmids Analysis

**Figure 5:**
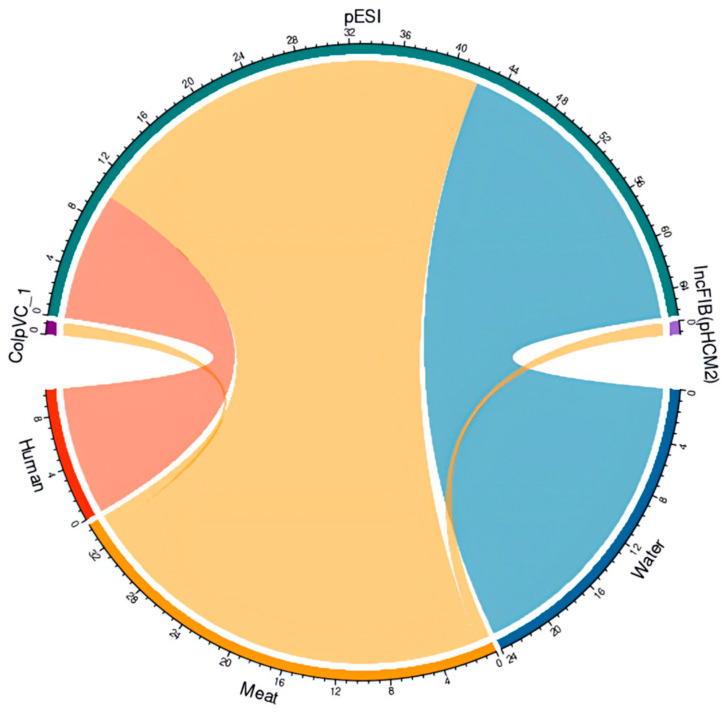
Plasmids detected in *Salmonella* Infantis isolates from humans, surface water, and poultry meat. Diagram of plasmids ColpVC_1 (1/73), IncFIB(pHCM2) (1/73), and pESI (plasmid of emerging *S.* Infantis) (69/73) detected in *S.* Infantis from humans (red, *n* = 11), surface water (blue, *n* = 30), and chicken meat (orange, *n* = 32). The circular graphs represent the plasmids identified in the *Salmonella* Infantis strains from human surface water and chicken meat, in green is pESI, in violet is IncFIB, and in purple is ColpVC.

We looked at the presence of plasmid replicons in *S.* Infantis isolates (*n* = 73) from all sources and identified different types of plasmids. The pESI-like plasmid was observed in 93% (69/73) of isolates (Figure 5 and Supplemental Table S5). We analyzed the pESI-like plasmid in a representative isolate (FA0496), based on raw sequencing reads, and identified the backbone regions of the megaplasmid, the presence of the qacED1 gene, the presence of class I integrons (intI genes, attC and attI regions), among other features (Supplemental Figure S2). Additional plasmids were observed in chicken meat isolates: ColpVC_1 [1% (1/73)] and IncFIB(pHCM2) plasmid [1% (1/73)] (Figure 5 and Supplemental Table S4).

## 4. Discussion

Non-typhoidal *Salmonella* remains a major food safety and public health concern, and the emergence of multidrug-resistant (MDR) lineages underscores the need for integrated One Health surveillance. In this study, WGS and antimicrobial susceptibility testing of poultry-meat isolates and a comparative analysis of *S.* Infantis from poultry meat, surface water, and human clinical cases revealed widespread resistance determinants and frequent pESI-like plasmid carriage, together with genomic relatedness across sources. These findings support circulation of MDR *S.* Infantis across connected reservoirs relevant to the poultry supply chain. Notably, closely related MDR *Salmonella* Infantis was detected in both clinical samples and surface waters, indicating high genetic similarity across sources. This genetic relatedness may suggest potential dissemination via contaminated poultry meat or environmental water sources; however, the direction and route of transmission cannot be determined from the present data. We acknowledge certain study limitations, including the short surveillance period (2018–2022) and the limited human sample size. The limited sampling size was due to sampling constraints, as isolates were obtained solely from one collaborative health center in Santiago, Chile. Including more human isolates could enhance the robustness of our analysis and potentially identify additional phylogenetic relations and possible unidentified outbreaks.

Given that poultry meat in Chile is supplied by both domestic production and imports, primarily from Brazil and Argentina [2]. The serovar composition and AMR profiles observed in our poultry-meat isolate collection highlight the potential for cross-border introduction and local circulation of resistant *Salmonella* lineages. In this isolate collection, serovar Enteritidis was detected in a single imported product from Argentina, while *S.* Minnesota was detected among imported products from Brazil and Argentina, and serovar Heidelberg was isolated in meats from all three countries. Overall, the serovar distribution observed in our collection is consistent with other studies showing similar *Salmonella* serovar distribution in chicken products across Latin American countries [1].

Shifts in *Salmonella* serovar distribution in the poultry industry have been reported worldwide. In Europe, *S.* Enteritidis prevalence in poultry meat is low; in Italy, the dominant serovars between 2011 and 2021 were *S.* Infantis, *S.* Derby, and monophasic *S.* Typhimurium [36]. In Brazil, studies report frequent detection of *S.* Minnesota and *S.* Heidelberg in broiler feces [37], and a rising prevalence of these serovars since 2014 in broilers, farms, and ready-to-eat chicken products [38]. In the United States, changes in *Salmonella* populations in poultry have been documented between 1996 and 2009, with decreasing *S.* Enteritidis and increasing *S.* Heidelberg and *S.* Kentucky [39]. Several researchers attribute these shifts to changes in chicken vaccination plans and the evolution and emergence of multidrug-resistant bacteria [39,40].

The detection of *bla*_CMY_ variants (including *bla*_CMY-2_ and *bla*_CMY-59_) among *S.* Heidelberg from poultry meat is consistent with prior reports linking these determinants to clinically relevant β-lactam resistance [37,41,42,43]. In Brazil, *S.* Heidelberg from chicken meat has been reported to exhibit multidrug resistance, including resistance to β-lactams and additional antimicrobial classes [37,41]. *S.* Heidelberg has also been isolated from human cases in Brazil [44], and global phylogenetic studies suggest chicken meat exported from Brazil could be a source of MDR *S.* Heidelberg [37,42,45]. In Chile, few reports exist, but *S.* Heidelberg was isolated from clinical and environmental samples between 2006 and 2011 [46], with one human isolate showing resistance to ceftriaxone and ceftiofur, ESBL production, and presence of *bla*_CMY-2_. Based on this evidence, *S.* Heidelberg may represent an emerging resistant pathogen with growing public health significance in the Chilean poultry supply.

Given the global emergence of MDR *S.* Infantis and its relevance to poultry-associated transmission, we compared *S.* Infantis from poultry meat with isolates from surface water and human clinical cases. The MDR patterns observed across these sources are consistent with those reported internationally for *S.* Infantis [17,47,48]. In Chile, previous studies have identified the presence of MDR *S.* Infantis isolates in poultry farms [30,49,50]. However, MDR *S.* Infantis dissemination is not limited to live poultry or poultry meat; it has also been isolated from human clinical cases, and environmental sources, including water [51]. These isolates pose a public health concern because they are frequently resistant to first and second-line antibiotics commonly used to treat salmonellosis [52]. This includes resistance to quinolones and third-generation cephalosporins, which are considered critical for public health and classified by the World Health Organization as critically important in human medicine [7]. The most common resistance reported in this study was to quinolone, followed by beta-lactams, aminoglycosides, and trimethoprim-sulfamethoxazole. Importantly, the highest frequency of detection of quinolone resistance was obtained from chicken meat isolates. Similarly, in other regions such as Europe, the Americas, the Western Pacific, and the Eastern Mediterranean, *S.* Infantis isolates from chicken meat also show quinolone resistance [53,54,55,56]. It could be a consequence of the use of these antibiotics in animal production [57]. For instance, in Chile, quinolones such as enrofloxacin, nalidixic acid, and ciprofloxacin are approved to treat infections in broiler production [58]. However, regulations for more controlled use in animal health are currently ongoing.

Human clinical *S.* Infantis isolates showed frequent third-generation cephalosporin non-susceptibility and ESBL-associated phenotypes, highlighting the clinical relevance of MDR lineages and ESBL determinants detected in this study. Overall, international reports in the European Union, Egypt, Japan, Chile, and the US showed increasing resistance to third-generation cephalosporins in isolates from human and chicken meat, and other samples (broiler, human, chicken meat, and environment), being isolates mainly of *S.* Infantis [18,54,59,60]. An ESBL phenotype was observed in *S.* Infantis isolates from broiler as well as human, chicken meat, and environmental sources, consistent with the presence of *bla*_CTX-M-65_ and *bla*_TEM-1_ among other resistance determinants [61,62]. Since 2019, the U. S. Food and Drug Administration has also observed a rise in resistance to ceftriaxone in isolates of *Salmonella* serovar I 4,[5],12: i:-from humans [63].

In this dataset, carbapenem non-susceptibility was not detected among human, surface water, or chicken meat isolates. Although carbapenem resistance in *Salmonella* spp. is uncommon [64,65], sporadic carbapenem-resistant *S.* Infantis has been reported (e.g., from a human clinical stool sample in Taiwan) [65]. These observations support the value of continued surveillance to detect the emergence of extensively drug-resistant *Salmonella* variants.

Across poultry meat, human clinical, and surface water sources, *S.* Infantis showed broadly similar AMR genotypes, including determinants consistent with resistance to aminoglycosides, phenicols (e.g., *floR*), fosfomycin (*fosA*), tetracyclines (*tet(A)*), sulfonamides/trimethoprim (*sul1*/*dfrA14*), and extended-spectrum cephalosporins (*bla*_CTX-M-65_), together with QRDR mutations associated with quinolone non-susceptibility (predominantly *gyrA* D87Y). This profile is consistent with pESI-associated MDR *S.* Infantis reported in broiler and human contexts in the Americas and Europe, including pESI-like lineages described from Ecuador, Peru, and the USA [14,66] and related resistance determinants reported from European poultry-associated isolates [14,67]. While the genomic relatedness observed across sources supports circulation among connected reservoirs, our data do not allow inference of directionality or confirmation that contaminated chicken meat was the primary route of dissemination. In addition, because detailed epidemiological linkage data were unavailable, the upstream origin(s) of MDR *S.* Infantis circulating in Chile cannot be determined from this study.

Quinolone resistance determinants showed source-dependent patterns in this dataset. Surface water isolates were phenotypically susceptible to ciprofloxacin and pefloxacin despite carrying the qnrB19 gene, consistent with the role of plasmid-mediated quinolone resistance (PMQR) in conferring low-level resistance and potentially facilitating selection of higher-level quinolone resistance [68]. In Chile, PMQR mediated by qnrB19 has been reported in multiple *Salmonella* serovars, including *S.* Enteritidis, *S.* Heidelberg, and *S.* Senftenberg, associated with a small plasmid harboring qnrB19 [69]. The qnrB19 gene has also been described beyond *S.* Infantis [70]. Moreover, recent data from Chile identified *S.* Infantis isolates from chicken meat carrying qnrB19 [49]. Together, these reports support the potential for horizontal transfer of qnrB19 among *Salmonella* serovars [71], and suggest that environmental and food-associated interfaces may contribute to its dissemination.

*S.* Infantis isolates from human clinical samples and chicken meat showed high genetic similarity [61]. Two genetically indistinguishable human clinical isolates and closely related chicken meat isolates were grouped within cluster eight. This finding raises the possibility of an undetected foodborne outbreak in 2019; however, epidemiological data were not available to substantiate this hypothesis.

Frequent carriage of pESI-like megaplasmids among *S. Infantis* supports the global pattern of MDR *S.* Infantis lineages reported across the Americas, Europe, and the Eastern Mediterranean [66,72,73]. The pESI-like features observed in this study, including ESBL-associated determinants (e.g., *bla*_CTX-M-65_), IncFIB(K) replicons, and QRDR mutations such as *gyrA* D87Y, are consistent with prior descriptions of emerging *S.* Infantis lineages [14,66]. Moreover, detection of *qacEΔ1*, a marker associated with tolerance to quaternary ammonium disinfectants, mirrors early pESI characterizations and suggests a potential contribution to persistence under hygiene practices relying on QAC-based disinfectants [74]. The presence of class I integrons (intI genes with attI/attC regions) within IncFIB(K) plasmid backbones is consistent with prior reports [75], and supports the role of pESI-like elements as platforms for the acquisition and dissemination of AMR-associated gene cassettes across diverse reservoirs. The representative pESI-like plasmid sequence also contained multiple mobile genetic element signatures (e.g., transposons and prophage-related regions) and genes associated with metal tolerance, including tellurite resistance determinants, which have been described in other Enterobacterales such as *E. coli* [76]. Together, these features are compatible with ongoing horizontal gene transfer and adaptive processes in environmental interfaces. In addition, detection of less common plasmid replicons (e.g., IncFIB(pHCM2)_1_pHCM2 and ColpVC_1) in a small subset of poultry-meat *S.* Infantis isolates suggests occasional acquisition of plasmid backbones more frequently reported in other serovars. IncFIB(pHCM2)-like replicons have been described in *S.* Tennessee recovered from dead chick embryos in Henan Province, China, during 2014–2015 (2139 embryos sampled across 28 hatcheries) [77] Additionally, a USA surveillance dataset from North Carolina (2018–2019) reported IncF-family virulence plasmid replicons (IncFII(S)_1 and/or IncFIB(S)_1) in poultry-associated serovars, including chicken-derived *S.* Enteritidis [78].

Chile-specific public health implications are notable. The detection of MDR *Salmonella,* including ESBL-associated determinants and fluoroquinolone resistance markers among poultry-meat isolates, supports prioritizing risk-based monitoring and preventive controls across domestic and imported poultry supply chains, coordinated with regional food safety oversight (SEREMI). Clinically relevant resistance determinants among human isolates underscore the importance of timely laboratory detection and reporting to inform empiric therapy and antimicrobial stewardship, particularly for invasive salmonellosis. The recovery of genetically related MDR *S.* Infantis from surface waters supports incorporating targeted environmental monitoring as a complementary component of national AMR surveillance. Collectively, these findings support an integrated “farm–food–environment–clinic” approach in Chile, in which WGS-informed characterization and coordinated data sharing between SEREMI-led food safety monitoring, clinical laboratories, and environmental programs can enable earlier detection, targeted monitoring of high-risk sources, and enhanced traceback when clusters span food and clinical isolates.

## 5. Conclusions

These findings support a One Health response to multidrug-resistant (MDR) *Salmonella* Infantis in Chile across poultry, human cases, and surface waters, while recognizing that transmission routes and directionality cannot be inferred from these data. The detection of closely related MDR *S.* Infantis in Chile, together with similar reports from other countries, indicates that this issue extends beyond a single national context.

Action is needed across three sectors. Human health: strengthen timely laboratory detection and reporting of key resistance determinants to guide empiric therapy and antimicrobial stewardship. Animal health and food safety: prioritize risk-based monitoring and preventive controls along domestic and imported poultry supply chains, with targeted follow-up when MDR lineages are detected. Environmental health integrates targeted surface-water monitoring into AMR surveillance to detect persistence and dissemination outside the food chain. Coordinated cross-sector surveillance and data sharing, supported by whole-genome sequencing (WGS), are essential for early detection and risk management.

## Figures and Tables

**Table 1:** Distribution of serovars for the 61 serovars recovered from poultry meat isolates collected in 2018.

*Salmonella* Serovars	No Isolates in Each Serovar (% of Total Isolates for Country)			
	Argentina (*n* = 3)	Brazil (*n* = 15)	Chile (*n* = 43)	Total (*n* = 61)
*S.* Agona	-	-	6 (14%)	6 (9.8%)
*S.* Enteritidis	1 (33.3%)	-	-	1 (1.6%)
*S.* Heidelberg	1 (33.3%)	9 (60%)	2 (5%)	12 (20%)
*S.* Infantis	-	1 (7%)	35 (81%)	36 (59%)
*S.* Minnesota	1 (33.3%)	4 (27%)	-	5 (8%)
*S.* Thompson	-	1 (7%)	-	1 (1.6%)

**Table 2:** Number and percentage of *S.* Infantis isolates that exhibit a phenotypic resistance to each antibiotic tested, indicating the antibiotic group, antibiotic name, and isolation source.

Antibiotic Group	Antibiotic	Number and Percentage of Isolates by Isolation Source			Total Number and Percentage of Isolates
		Poultry Meat	Human	Water	
Beta-lactams	Ampicillin	56% (18/32)	82% (9/11)	80% (24/30)	70% (51/73)
	Ampicillin/sulbactam	3% (1/32)	19% (1/11)	-	3% (2/73)
	Cefazoline	88% (28/32)	82% (9/11)	80% (24/30)	84% (61/73)
	Cefuroxime	56% (18/32)	82% (9/11)	77% (23/30)	68% (50/73)
	Cefotaxime	53% (17/32)	82% (9/11)	80% (24/30)	68% (50/73)
	Ceftazidime	-	19% (1/11)	70% (21/30)	30% (22/73)
	Cefixime	56% (18/32)	82% (9/11)	80% (24/30)	70% (51/73)
	Cefepime	-	19% (1/11)	-	1% (1/73)
Aminoglycoside	Gentamycin	56% (19/32)	73% (8/11)	47% (14/30)	56% (41/73)
Sulfonamide	Trimethoprim-Sulfamethoxazole	56% (18/32)	45% (5/11)	43% (13/30)	49% (36/73)
Quinolone	Pefloxacin	100% (32/32)	91% (10/11)	87% (26/30)	93% (68/73)
	Ciprofloxacin	100% (32/32)	91% (10/11)	87% (26/30)	93% (68/73)

**Table 3:** Presence of horizontally acquired genes (harboring a megaplasmid emergent (pESI-like)) in *Salmonella* Infantis isolates from poultry meat, humans and water surface.

Antibiotic Group	Horizontally Transmitted Genes	Number and Percentage of Isolates by Isolation Source			Total Number and Percentage of Isolates
		Poultry Meat	Human	Water	
Aminoglycoside	aac(3)-IVa	91% (29/32)	91% (10/11)	83% (25/30)	88% (64/73)
	*ant(3″)-Ia*	-	91% (10/11)	-	14% (10/73)
	*aph(3′)-Ia*	-	36% (4/11)	-	5% (4/73)
	*aph(3′)-Ic*	44% (14/32)	-	83% (25/30)	53% (39/73)
	*aph(4)-Ia*	84% (27/32)	91% (10/11)	83% (25/30)	85% (62/73)
	*aadA1*	97% (31/32)	-	90% (27/30)	79% (58/73)
Beta-lactam	*bla*_CTX-M-65_	53% (17/32)	82% (9/11)	83% (25/30)	70% (51/73)
Trimethoprim	*dfrA14*	53% (17/32)	55% (6/11)	57% (17/30)	55% (40/73)
Chloramphenicol	*floR*	81% (26/32)	91% (10/11)	83% (25/30)	84% (61/73)
Fosfomycin	*fosA*	38% (12/32)	82% (9/11)	30% (9/30)	41% (30/73)
Sulfonamide	*sul1*	97% (31/32)	91% (10/11)	90% (27/30)	93% (68/73)
Tetracycline	*tet(A)*	97% (31/32)	91% (10/11)	90% (27/30)	93% (68/73)
Quinolone	*qnrB19*	-	45% (5/11)	-	7% (5/73)

**Table 4:** Presence of chromosomal mutations in *Salmonella* Infantis isolates from poultry meat, humans, and water surface.

Antibiotic Group	Chromosomal Points Mutations	Number and Percentage of Isolates by Isolation Source			Total Number and Percentage of Isolates
		Poultry Meat	Human	Water	
Quinolone	*gyrA* (D87Y)	91% (29/32)	91% (10/11)	90% (27/30)	90% (66/73)
	*gyrB* (S464Y)	-	19% (2/11)	-	3% (2/73)
	*gyrA* (S83F)	-	9% (1/11)	-	1% (1/73)

## Data Availability

The original contributions presented in the study are included in the article/Supplementary Materials. Further inquiries can be directed to the corresponding author.

## Supplementary Materials

The following supporting information can be downloaded at: https://www.mdpi.com/article/10.3390/foods15030410/s1, Figure S1: Phylogenetic analysis. A. Phylogenetic tree based on core-genome Single Nucleotide Polymorphisms (cgSNPs) of ten *S.* Heidelberg strains from poultry meat isolates. B. Phylogenetic tree based on core-genome Single Nucleotide Polymorphisms (cgSNPs) of 37 *S.* Infantis strains from poultry meat isolates. The legend indicates the source of origin and year of isolation. Additionally, resistance genes are indicated for each isolate. The resistant genes are grouped by antibiotic class (blue ocean color: aminoglycosides; red: beta-lactam; yellow: trimethoprim; orange: florfenicol; fuchsia: fosfomycin; violet: sulphonamides; blue, cerulean: tetracyclines; green lime: quinolones). Figure S2: Representation of the resistant pattern of pESI-like from a surface water sample (FA0496). We identified antibiotic-resistant genes related to aminoglycosides, beta-lactams, tetracycline, sulfonamides, and florfenicol. Additionally, we detected resistance genes for quaternary ammonium, mercury, and tellurite. Table S1: Initial isolates of *Salmonella* spp. detected from poultry meat, water surface, and human samples. Table S2: Selected isolates of *Salmonella* spp. detected from poultry meat, water surface, and human samples. Table S3: Minimal Inhibition Concentration Breakpoints used in antimicrobial susceptibility test. Table S4: Phenotypic and genotypic resistance profile from poultry meat, water surface, and human clinical isolates. Table S5: Plasmids detected in poultry meat, water surface, and human clinical isolates.
