# Supplementary material for: Tracking Antimicrobial Resistance in Salmonella via Poultry Supply Chains, Human Clinical Samples, and Environmental Reservoirs
Source: Foods. 2026 Jan 23;15(3):410. doi: 10.3390/foods15030410 (PMC12897130; doi:10.3390/foods15030410)
Supplement: Supplementary file 1 [file foods-15-00410-s001.zip › Supplementary_Material_Captions_4033248.pdf]

Supplemental tables

- **Supplemental Table S1**: Initial isolates of *Salmonella* spp. detected from poultry meat, water surface, and human samples.

- **Supplemental Table S2**: Selected isolates of *Salmonella* spp. detected from poultry meat, water surface, and human samples.

- **Supplemental Table S3:** Minimal Inhibition Concentration Breakpoints used in antimicrobial susceptibility test.

- **Supplemental Table S4:** Phenotypic and genotypic resistance profile from poultry meat, water surface, and human clinical isolates.

- **Supplemental Table S5:** Plasmids detected in poultry meat, water surface, and human clinical isolates.

Supplemental figures

Supplemental figure S1. Phylogenetic analysis. A. Phylogenetic tree based on core-genome Single Nucleotide Polymorphisms (cgSNPs) of ten *S.* Heidelberg strains from poultry meat isolates. **B.** Phylogenetic tree based on core-genome Single Nucleotide Polymorphisms (cgSNPs) of 37 *S.* Infantis strains from poultry meat isolates. The legend indicates the source of origin and year of isolation. Additionally, resistance genes are indicated for each isolate. The resistant genes grouped by antibiotic class (blue ocean color: aminoglycoside; red: beta-lactam; yellow: trimethoprim; orange: florfenicol; fuchsia: fosfomycin; violet: sulphonamides; blue, cerulean: tetracycline; green lime: quinolone).

Supplemental Figure S2. **Representation of the resistant pattern of pESI-like from a surface water sample (FA0496).** We identified antibiotic-resistant genes related to aminoglycoside, beta-lactam, tetracycline, sulfonamides, and florfenicol. Additionally, we detected resistance genes for quaternary ammonium, mercury, and tellurite.
