# Supplementary figures and images for: Tracking Antimicrobial Resistance in Salmonella via Poultry Supply Chains, Human Clinical Samples, and Environmental Reservoirs
Source: Foods. 2026 Jan 23;15(3):410. doi: 10.3390/foods15030410 (PMC12897130; doi:10.3390/foods15030410)

Tree scale: 0.001

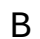

Tree scale: 0.01

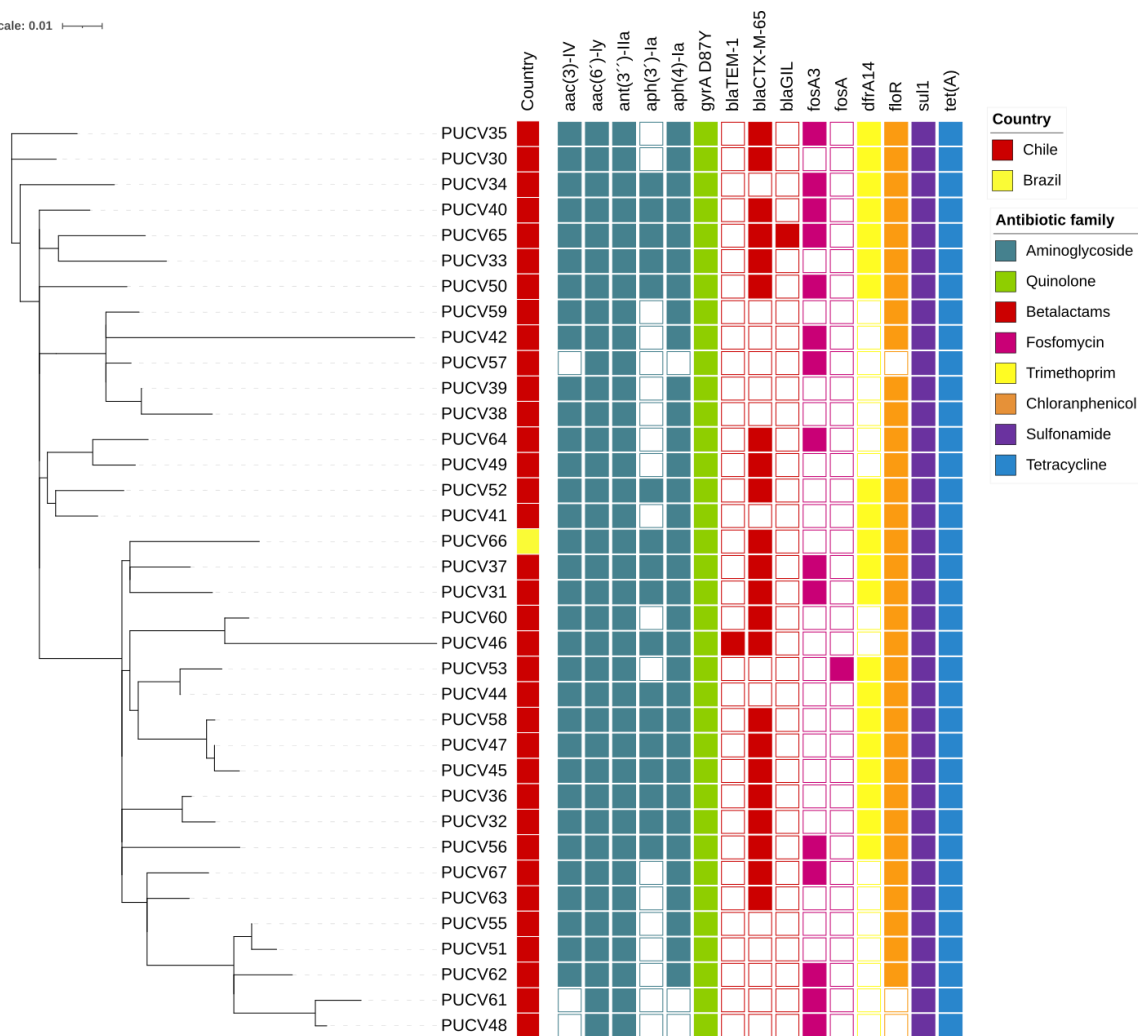

Supplement: Supplementary file 1 [file foods-15-00410-s001.zip › Supplementary Figure S1.pdf]

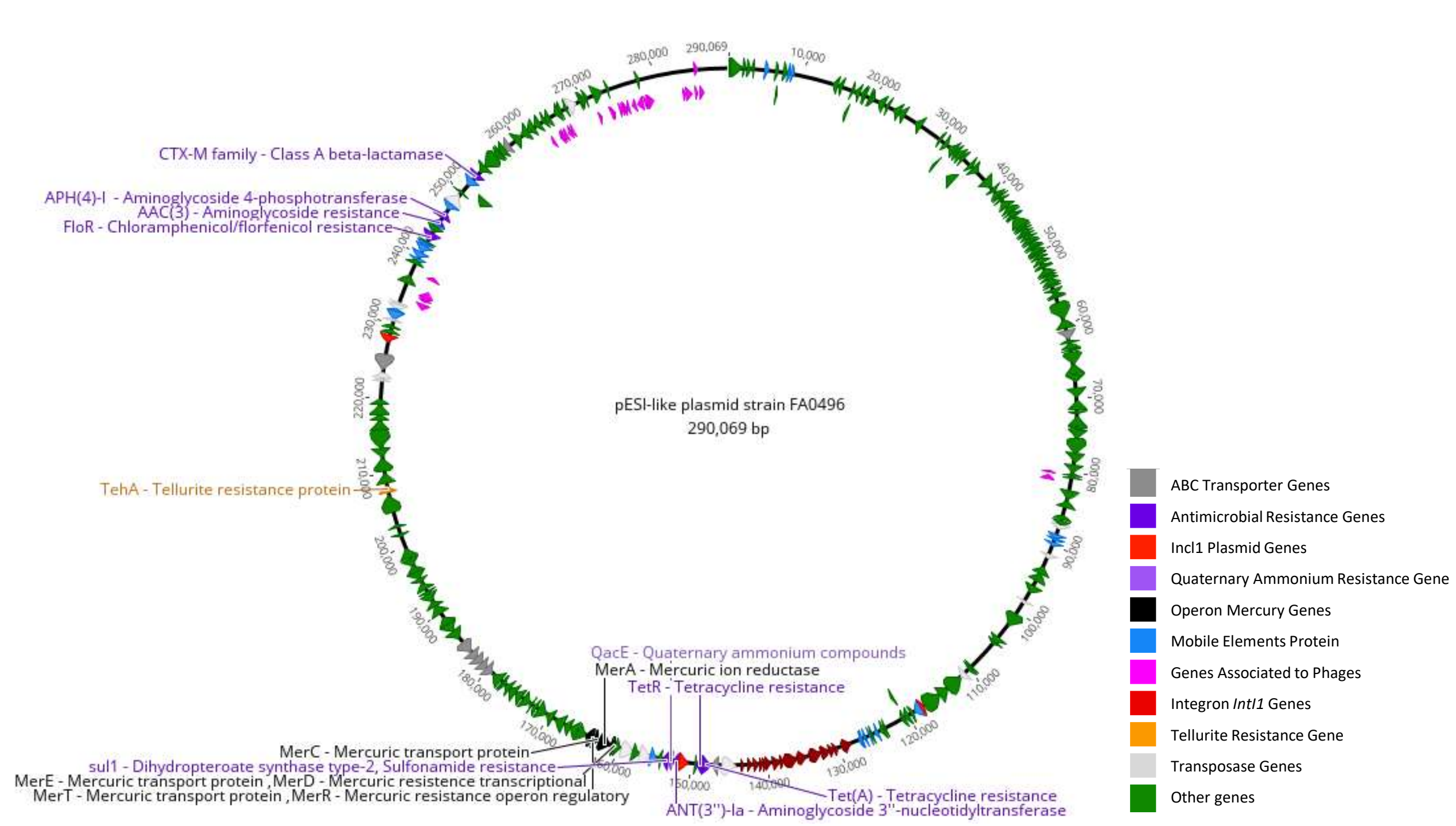

Supplement: Supplementary file 1 [file foods-15-00410-s001.zip › Supplementary Figure S2.pdf]
